# Serological evidence of bluetongue virus infection and serotype distribution in dairy cattle in South Korea

**DOI:** 10.1186/s12917-019-2000-z

**Published:** 2019-07-23

**Authors:** Jeong-Min Hwang, Jae Geun Kim, Jung-Yong Yeh

**Affiliations:** 1 Veterinary Research Center, Green Cross Veterinary Products Co., Ltd, Kugal-dong 227-5, Giheung-gu, Yongin-si, Gyeonggi-do 17066 South Korea; 2 Department of Life Sciences, College of Life Sciences and Bioengineering, Incheon National University, Academy-ro 119, Yeonsu-gu, Incheon, 22012 South Korea; 3 Emerging & Exotic Diseases Research Laboratory, Foreign Animal Diseases Division, National Veterinary Research and Quarantine Service, Anyang-ro 175, Manan-gu, Anyang-si, Gyeonggi-do 14089 South Korea

**Keywords:** Bluetongue, Seroprevalence, Serotype, Dairy cattle, South Korea

## Abstract

**Background:**

Bluetongue is a vector-borne viral disease, and bluetongue virus (BTV) outbreaks can cause substantial economic losses. Even subclinical infection may carry significant associated costs, including a loss of condition, reduced milk yield, and infertility and abortion, and indirect costs, largely due to the export restrictions and surveillance requirements imposed to limit the spread of the virus. However, the BTV epidemiology in the Far East remains incompletely understood, especially in the cattle population in South Korea. In this study, the seroprevalence of BTV antibodies and distribution of BTV serotypes in dairy cattle in South Korea were evaluated to improve the understanding of the BTV epidemiological situation in the Asia-Pacific region.

**Results:**

Between 2012 and 2013, a total of 37 out of 171 dairy cattle herds (21.6%) and 85 out of 466 dairy cattle heads (18.2%) showed antibodies against BTV. Neutralizing antibodies to BTV-1, − 2, − 3, − 4, − 7, − 15, and − 16 serotypes were identified, and the RNAs of the BTV-1, − 2, − 3, − 15, and − 16 serotypes were detected, indicating that BTV was circulating in the dairy cattle population in South Korea.

**Conclusions:**

These findings indicate that BTV is widespread and has circulated in dairy cattle in South Korea. This is the first report presenting evidence of circulating antibodies against BTV and the serotype distribution in bovine populations in South Korea.

## Background

Bluetongue is a vector-borne viral disease that affects wild and domestic ruminant species and reduces herd productivity [1]. Even subclinical infection may carry significant associated costs, including a loss of condition, reduced milk yield, and infertility and abortion [2], and indirect costs, which largely arise from the export restrictions and surveillance requirements imposed to limit the spread of the bluetongue virus (BTV) [3]. Because it can spread rapidly and cause serious economic consequences in affected countries, bluetongue has been classified as a notifiable disease by the World Organization for Animal Health (OIE) [4].

Bluetongue in sheep is characterized by fever, facial edema, hyperemia, congestion, and erosion of mucous membranes. Bluetongue disease results from vascular injury, likely through a process analogous to that of human hemorrhagic viral fevers, in which the production of vasoactive mediators from virus-infected macrophages and dendritic cells results in enhanced endothelial paracellular permeability with subsequent vascular leakage and hypovolemic shock [5]. BTV infections in ruminants can be subclinical, though fatal diseases predominantly occur in sheep, deer, and wild ruminants [6, 7].

Although bluetongue was recognized and described more than 100 years ago in southern Africa, BTV infection of ruminants, as an enzootic disease, and its vector, *Culicoides* (Diptera: *Ceratopogonidae*) genus insects are traditionally found in tropical and temperate regions of the world between the latitudes of approximately 40° North and 35° South. Exceptions include regions of Asia and western North America, where BTV infection of ruminants occurs as far as 50° North [5, 8–10]. However, there have been drastic recent regional alterations in the global distribution of BTV infection, particularly in Europe, since 1998 [5]. BTV is considered an emerging disease in Europe, where the disease has spread with unprecedented speed and virulence. There is a substantial body of evidence linking this emergence to climate change [3], and it has been proposed, but certainly not proven, that global climate change is responsible for these events [5].

The epidemiology of BTV infection is poorly defined in much of the world, including extensive portions of Asia. In Asia, bluetongue was first recognized in Israel in 1949 and was later recognized in the People’s Republic of China in 1979 [11], Indonesia in 1981 [12], Japan in 1985 [13], and Malaysia in 1987 [14]. Although the importance of BTV as a transboundary and emerging disease in the world has been suggested, the epidemiology of BTV in South Korea and its neighboring countries in the Asia-Pacific region remains unclear. In the Far East, evidence of BTV has been found in countries neighboring South Korea. It has been accepted that BTV may be endemically established in Japan and China [13, 15–28], and epidemiological studies of the wildlife in Japan have noted the current existence of interconnected domestic and wild cycles that could account for the maintenance of BTV [29]. There was no BTV outbreak or case report in South Korea until 2015, when BTV was isolated from whole-blood samples taken from cattle at abattoirs [30]. Because there was no information on BTV in cattle in South Korea, a study was designed to evaluate the seroprevalence of BTV antibodies and distribution of BTV serotypes in dairy cattle to improve the understanding of the epidemiological situation of BTV in the Asia-Pacific region.

## Results

**Fig. 1:**
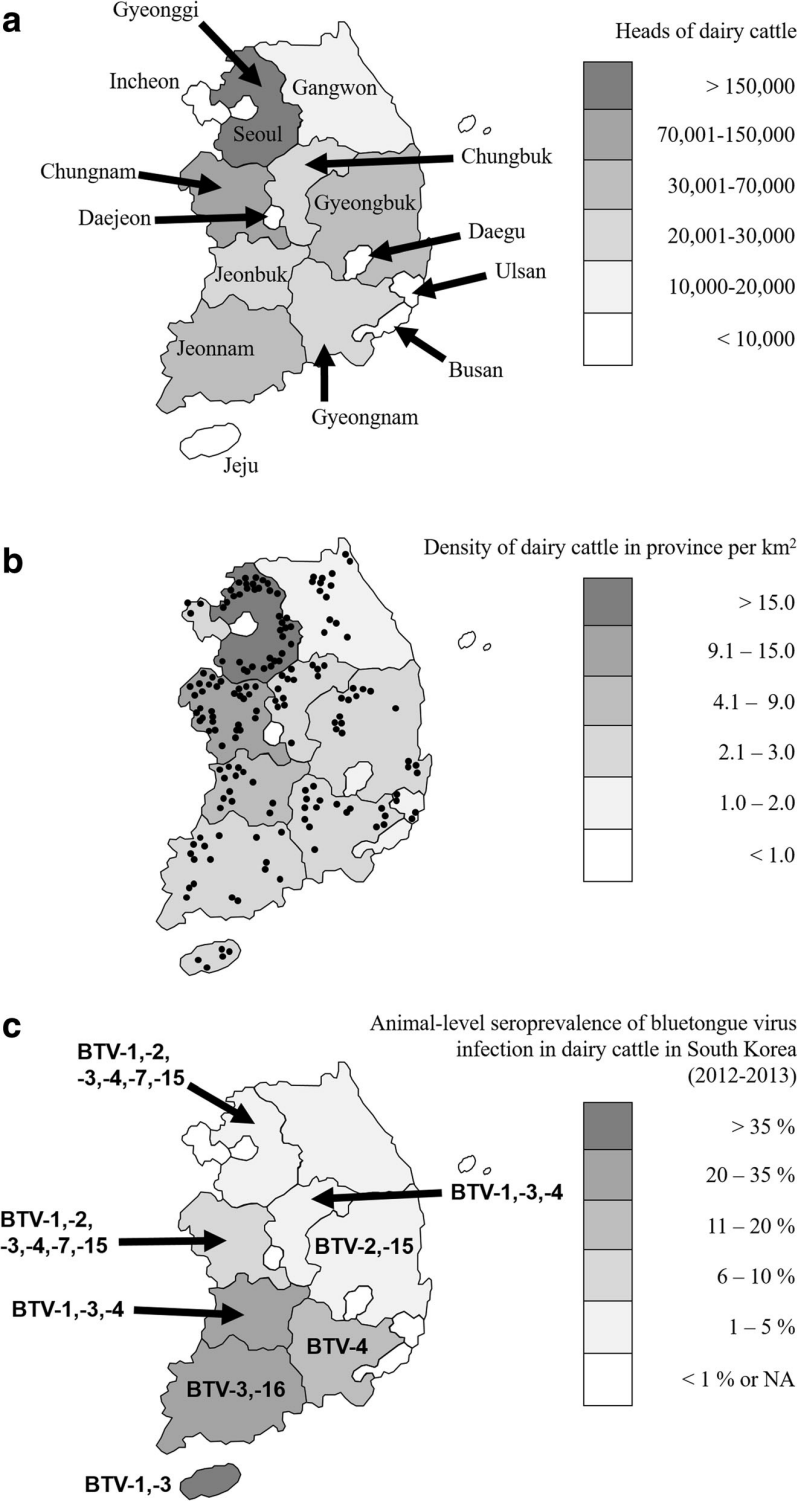
**a** Geographical location of the provinces in South Korea. **b** Density of dairy cattle in South Korea per km^2^. The herds sampled in this study are mapped by a dot. **c** Geographical distribution patterns of the seroprevalence to BTV. Percentages of tested animals seropositive for bluetongue virus in 11 provinces in South Korea. The serotypes identified by serum neutralization tests or the BTV serotype identified by RT-PCR of blood sample are shown. Differences in the intensity of the darkness on the map represent different percentages of seroprevalence. The map depicted in figure 1 is our own

**Fig. 2:**
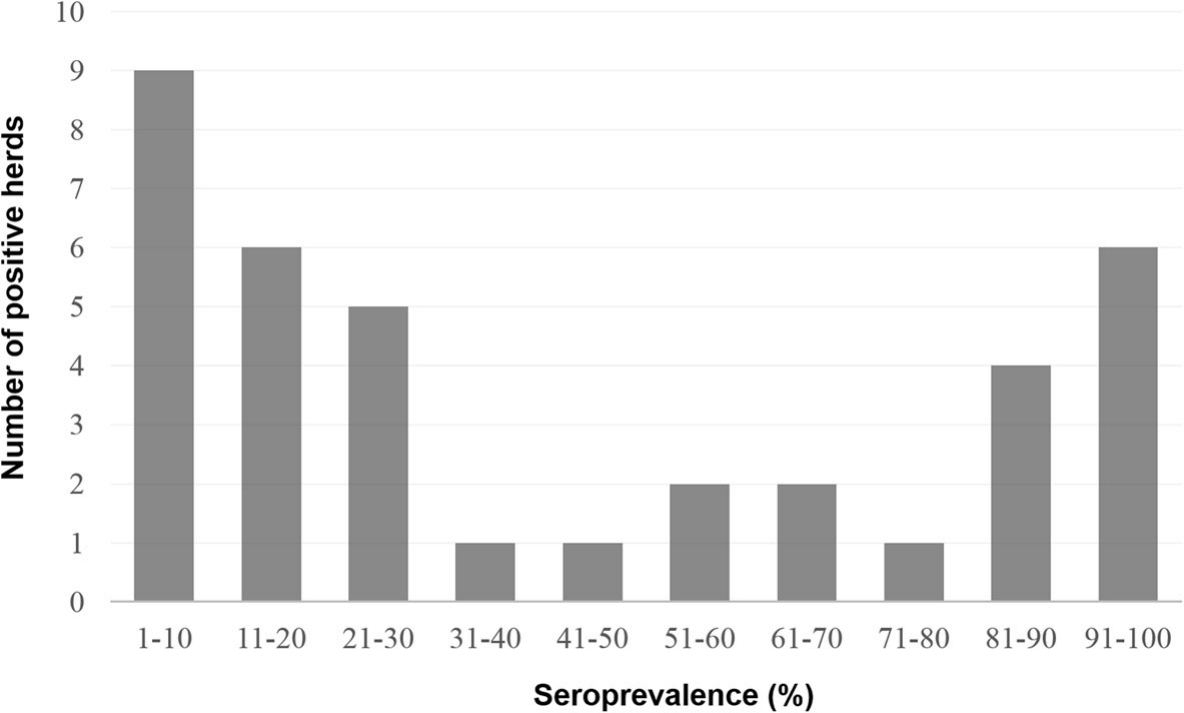
Frequency distribution of the seroprevalence proportions of bluetongue virus infection in dairy cattle in South Korea (2012–2013). Note the bimodal distribution; one cluster is < 30% and the other cluster > 81% among individual cattle herds

Between 2012 and 2013, at the national level, 37 of 171 dairy cattle herds (21.6, 95% confidence interval: 16.1–28.4%) analyzed and 85 of 466 dairy cattle heads (18.2, 95% confidence interval: 15.0–22.0%) analyzed showed antibodies against BTV using cELISA as shown in Table 1. The agreement of the serologic status between dairy cattle sampled within the same herd in our prevalence study, as measured by the intra-class correlation coefficient, was 0.21. Our results identified the population sizes of herds to be a protective factor. An increased number of animals inside the farm led to a decreased risk of being positive. In the univariable analysis (Table 2), no significant risk differences in land use, adult/calf ratios, and experience of reproductive problems were found. Our results identified herd size as a protective factor. An increased number of animals inside the farm led to risk of being positive (OR = 2.762, *p* = 0.003 in a herd of ≤5 animals; OR = 4.174, *p* = < 0.001 in a herd of 6–30), while older age was shown to be a significant risk factor (OR = 0.294, *p* = < 0.001 in juveniles; OR = 0.443, *p* = 0.004 in a herd of subadults). The cattle density for the class of 11–20 animals was another significant risk factor (OR = 1.737, *p* = 0.070), while cattle densities for the classes of ≤10 or ≥ 21 were not significantly associated. We observed a significant difference in the individual likelihood of being positive in southern provinces with respect to northern provinces (OR = 1.757, *p* = 0.019). A significant difference was also observed in the individual likelihood of being positive in western provinces with respect to eastern provinces (OR = 0.531, *p* = 0.026). The risk factors identified in the multivariable logistic model (*p* < 0.001) were as follows: older animals (adults), southern, and northern provinces. The herd size was confirmed to be a protective factor (Table 3). Of the positive dairy cattle herds, 82.4% (28/34) were clustered in either < 30% or > 81%, suggesting a bimodal frequency distribution (Fig. 2). Additionally, there were substantial regional differences in the seroprevalence within South Korea (Fig. 1). Of the 85 ELISA-positive samples, only 59 were positive by SNT (Table 1) and neutralized one or more BTV serotypes: 1 (26 serum samples), 2 (11 serum samples), 3 (15 serum samples), 4 (13 serum samples), 7 (6 serum samples), 15 (9 serum samples), and 16 (13 serum samples). These findings support the notion that BTV-1 is the most prevalent serotype in South Korea. By contrast, 26 dairy cattle serum samples failed to neutralize any known BTV serotype. Additionally, the RNA of the BTV-1, − 2, − 3, − 15, and − 16 serotypes was detected in 13 serologically positive blood samples by RT-PCR, indicating that several BTV serotypes were actively circulating in the dairy cattle populations in the studied area. Further phylogenetic analysis and virus isolation for these blood samples could not be performed using these samples because of an insufficient blood volume and quality of the positive samples.

**Table 1:** Seroprevalence of BTV infection in dairy cattle in the Republic of Korea (2012–2013)

			Dairy cattle (herds)			Dairy cattle (individuals)				Serotype	
Province	Latitude (N)	Longitude (E)	Positive^a^	Tested	AP^b^	Positive	Tested	AP	TP ± 95% CI	SNT^d^	RNA (number)^e^
Incheon	36°55'-37°58'	124°36'-126°47'	0	3	0	0	9	0	0 - 29.9	-	-
Ulsan	35°19'-35°43'	128°58'-129°27'	0	4	0	0	12	0	0 - 24.3	-	-
Gyeonggi	36°53'-38°17'	126°22'-127°51'	7	37	18.9	18	109	16.5	10.7 - 24.6	1, 2, 3, 4, 7, 15	2 (2), 15 (1)
Gangwon	38°09'-39°25'	126°46'-128°22'	0	14	0	0	31	0	0 - 11.0	-	-
Chungbuk	37°15'-36°00'	127°16'-128°38'	2	14	14.3	3	33	9.1	3.1 - 23.6	1, 4	1 (1), 3 (1)
Chungnam	35°58'-37°03'	125°32'-127°38'	8	32	25.0	19	111	17.1	11.2 - 25.2	1, 2, 3, 4, 7, 15	2 (1), 3 (2), 15 (1)
Jeonbuk	35°18'-36°09'	125°58'-127°54'	6	13	46.2	14	37	37.8	24.1 - 53.9	1, 3, 4	ND^f^
Jeonnam	33°54'-35°30'	125°04'-127°54'	7	17	41.2	17	41	41.5	27.8 - 56.6	3, 16	16 (1)
Gyeongbuk	35°34'-37°33'	127°48'-131°52'	2	16	12.5	4	34	11.8	4.7 - 26.6	2, 15	2 (1)
Gyeongnam	34°39'-35°54'	127°35'-129°28'	3	16	18.8	5	36	13.9	6.9 - 28.7	4	ND^f^
Jeju	33°06'-34°00'	126°08'-126°58'	2	5	40.0	5	13	38.5	17.7 - 64.5	1, 3	1 (1), 3 (1)
Total	33°06'-39°25'	124°36'-131°52'	37	171	21.6	85	466	18.2	15.0 - 22.0		

^a^Number of seropositive herds or animals (individuals); ^b^AP, apparent (estimated) prevalence; ^c^TP ± 95% CI, 95% confidence interval for the true proportion; ^d^Serotypes identified by serum neutralization tests; ^e^BTV serotype identified by RT-PCR for blood sample; ^f^ND, not detected

**Table 2:** Univariable analysis of the exposure variables relative to the animal seropositivity outcome

	Negative (*N*=381)	Positive (*N*=85)	OR (95% CI)	*p* value
Land use				
Agricultural	117	30	1.262 (0.705 - 2.259)	0.432
Woodland and semi-natural	136	29	1.050 (0.587 - 1.878)	0.870
Urban	128	26	-	-
Herd size				
≤5	122	30	2.762 (1.380 - 5.527)	0.003
6-30	113	42	4.174 (2.139 - 8.148)	<0.001
≥31	146	13	-	-
Cattle density				
≤10	120	30	1.625 (0.876 - 3.014)	0.121
11-20	131	35	1.737 (0.953 - 3.166)	0.070
≥21	130	20	-	-
Adult/calf ratios				
≤1.0	135	20	0.653 (0.348 - 1.224)	0.182
1.1-2.0	127	38	1.319 (0.759 - 2.293)	0.326
≥2.1	119	27	-	-
Age class				
Juvenile	145	18	0.294 (0.159 - 0.541)	<0.001
Subadult	139	26	0.443 (0.254 - 0.771)	0.004
Adult	97	41	-	-
Reproductive problems				
Yes	173	41	1.120 (0.700 - 1.794)	0.636
No	208	44	-	-
Southern vs northern				
Southern	253	45	1.757 (1.092 - 2.828)	0.019
Northern	128	40	-	-
Eastern vs western				
Eastern	128	18	0.531 (0.303 - 0.932)	0.026
Western	253	67	-	-

**Table 3:** Multivariable analysis of the exposure variables relative to the animal seropositivity outcome

Variable	OR	95% CI	*p* value
Land use			
Agricultural	1.446	0.751 - 2.784	0.270
Woodland and semi-natural	1.034	0.542 - 1.973	0.920
Urban	-	-	-
Herd size			
≤5	2.951	1.411 - 6.171	0.004
6-30	4.512	2.218 - 9.180	<0.001
≥31	-	-	-
Cattle density			
≤10	1.478	0.745 - 2.935	0.264
11-20	1.711	0.883 - 3.314	0.111
≥21	-	-	-
Adult/calf ratios			
≤1.0	0.578	0.291 - 1.150	0.118
1.1-2.0	1.550	0.841 - 2.857	0.160
≥2.1	-	-	-
Age class			
Juvenile	0.249	0.128 - 0.484	<0.001
Subadult	0.449	0.244 - 0.828	0.010
Adult	-	-	-
Reproductive problems			
Yes	1.081	0.641 - 1.822	0.771
No	-	-	-
Southern vs northern			
Southern	3.525	2.000 - 6.214	<0.001
Northern	-	-	-
Eastern vs western			
Eastern	0.332	0.171 - 0.647	0.001
Western	-	-	-

## Discussion

Our study provides evidence for the serological prevalence of circulating antibodies against BTV and RNA of the BTV-1, − 2, − 3, − 15, and − 16 serotypes in the dairy cattle population in South Korea. The present study demonstrated that BTV infection was prevalent in the dairy cattle populations analyzed, in which approximately one in five dairy cattle herds and one in six dairy cattle were infected (Table 1). Further studies might include virological and serological investigations of BTV-4 and -7 circulating in South Korea because neutralizing antibodies against BTV-4 and -7 have been detected in dairy cattle serum samples. However, none of the blood samples in the present study showed virological evidence of BTV-4 and -7. To understand the results of the present study, it is important to know that South Korea has no vaccination program for bluetongue. Thus, the high seroprevalence of BTV infection in South Korea can be assumed to reflect a natural infection of the dairy cattle evaluated.

Briefly, with respect to Asian countries and the Middle East, the overall seroprevalence obtained was lower than that previously reported in this species in Taiwan, Nepal, India, and Japan [31–34], but higher than that previously reported in central and south-east Iran [35, 36]. The seroprevalence of BTV infection described in our study (18.2%) is comparable to that described among sentinel ruminants, such as cattle, buffalo, and goats, in China (17.1% seroprevalence) [37] and Indonesian ruminants (2–23% seroprevalence) [38]. The seroprevalence determined in this study is lower than that described among ruminants in Taiwan (32.7% in cattle and 8.2% in goats) [31], sentinel cattle in Japan (5–71%) [13], and sheep and cattle in Israel (16.7% in sheep and 63.2% cattle) [39] but higher than that reported in domestic yaks in China (2–5%) [23].

It was noted that 81.1% (28/34) of the seropositive dairy cattle herds clustered at either < 30% or > 81%, suggesting a “bimodal” distribution (Fig. 2), similar to that described by Taiwanese and Dutch research groups [31, 40]. The total dairy cattle population of South Korea is mostly composed of the Holstein breed. In 2015, the dairy cattle population in South Korea was composed of 402,405 bovines, which were kept on 5,407 holdings (approximately 74 dairy cattle per holding) [41]. The highest density of dairy cattle was in Gyeonggi Province, with 15.7 dairy cattle per km^2^ (Fig. 2). The lack of clinical signs of BTV might also be because the development of bluetongue in sheep has attracted little attention in South Korea because of the very small number of domestic sheep. Consequently, most BTV episodes throughout the world may be completely silent. Therefore, the results of the seroepidemiological surveillance performed in this study suggest that subclinical or mild BTV infection of ruminants in South Korea is prevalent almost every year and that there are repeated and recurrent infections among domestic cattle and other ruminants in the affected regions.

Despite the serological evidence of BTV infection and the serotype distribution in dairy cattle, there is no clinical report of bluetongue in any species in South Korea. BTV is apparently still infecting dairy cattle but causing subclinical disease. The factors related to the lack of a routine monitoring system, difficulties in making a clinical diagnosis, and misdiagnoses of similar viral diseases should also not to be ignored. For example, Aino virus, Akabane virus, Chuzan virus, and Ibaraki virus present the greatest difficulty for differential diagnosis because these viral infections cause similar symptoms and have high seropositive rates in sentinel cattle in South Korea, 33.2% for Aino virus, 40.2% for Akabene virus, 29.1% for Chuzan virus, and 7.5% for Ibaraki virus [42]. In the present study, an intra-class correlation coefficient of 0.21 was found, indicating that the correlation between two animals within a herd with respect to the BTV result was relatively high. The intra-class correlation coefficient of livestock infectious disease is usually < 0.2 and ranges from 0.04 to 0.42 [43]. This finding indicates that in any particular dairy cattle herd, it is likely that either most dairy cattle in that herd will be serologically positive or most will be negative. Our results revealed a significant association between age and BTV seroprevalence in South Korea, and a similar association was previously reported by several groups studying other cattle populations [44–46]. This association between age class and BTV seropositivity can be explained by a longer time of exposure for adults [46, 47]. The proportion of seropositive animals increased with age, probably resulting more from prolonged exposure of the adults to the vector than to any resistant status of juveniles. BTV infection significantly decreased with the increase in the number of dairy cattle inside a farm. The virus pressure in the vectors and ruminants would decrease due to the dilution effect caused by the increase in the density of susceptible dairy cattle on the farm. Nevertheless, this interesting finding requires further investigation. The results obtained from this study indicate that northern and eastern regions are areas of relatively low BTV seroprevalence. Relative to the factors that affect the likelihood of infection at an individual animal level, living in southern and western regions in South Korea increased the risk of being BTV positive compared with living in northern and eastern areas. This result could partially be explained by the spatial distribution of the *Culicoides* group, but the association between the BTV epidemiology and *Culicoides* vector distribution in South Korea is not fully understood.

## Conclusions

Collectively, in this study, the significance of the serological evidence and the findings of BTV serotypes distributed in dairy cattle South Korea are discussed and further studies to isolate local BTV strains from sentinel animals, including dairy cattle, are proposed. Entomological surveillance of biting *Culicoides* midges has been consistently conducted in South Korea since 2010 [48–52], and an epidemiological analysis is being assessed based on the vector species abundance, diversity, and competence of *Culicoides* midges distributed in South Korea. In addition, future surveillance programs for BTV should be extended to include other susceptible animals, such as sheep, cattle, and other ruminants. To our knowledge, this is the first report of evidence of circulating antibodies against BTV and the serotype distribution in the bovine population in South Korea. These findings indicate that BTV is widespread and actively circulating in dairy cattle in South Korea. The results also support the idea that serosurveillance of this species would be useful for detecting BTV circulation [34, 53–56]. Thorough research on the hosts and vectors involved in BTV circulation in natural ecosystems in the Far East is also needed, as has previously been suggested elsewhere [30].

## Methods

### Field samples for survey of BTV serology and the serotype distribution

A serological survey was conducted between 2012 and 2013 on dairy cattle for specific antibodies to BTV. Dairy cattle were selected as the target population because seroconversions usually occur earlier in bovine species (they are the preferred host [57]) and BTV antibody prevalence is considered to be higher in cattle (49–60%) than in sheep or goats (13.7–20%) [44, 58–62]. For this study, serum from as many animals as possible was collected from each herd based on the estimated seroprevalence of 10% and a confidence limit of 95% [63]. Herds and animals within the herds were selected by a simple random sampling method in each province based on the government’s national statistics [64]. The sampling frame was established using the dairy cattle farm ID and herd size obtained from the KAHIS (Korea Animal Health Integrated System, Animal and Plant quarantine agency, Anyang, South Korea). Animals younger than 6 months were excluded from the sampling frame to avoid the detection of antibodies due to maternal immunity. The apparent prevalence rates were considered to be the animal-level prevalence, defined as the proportion of cELISA-positive animals out of the total number of animals tested in the study area, and the herd prevalence, which was the proportion of cELISA-positive herds out of the total number of tested herds in the area. A herd was classified as positive if at least one animal was found to be cELISA positive. The intra-class correlation coefficient μ was calculated to measure the serologic status agreement between dairy cattle sampled within the same herd. Samples from dairy cattle were obtained from the blood and serum bank of the National Surveillance Program maintained by the Foreign Animal Diseases Division of the National Veterinary Research and Quarantine Service (Anyang, South Korea) in close collaboration with local veterinary practitioners and/or government veterinary officers. The number of samples from each province is shown in Table 1. The serum separated from the blood samples was stored at − 20 °C until further analysis. The seroprevalence rates were estimated at the herd and animal levels in dairy cattle, and the study was conducted in South Korea (33°06′ N - 39° 25′ N, 124°36′ E - 131°52′ W) from 2012 through 2013.

### Determination of BTV antibodies

It was previously reported that specific serodiagnostic techniques, such as competitive enzyme-linked immunosorbent assay (cELISA) or BTV neutralization tests, should be used for bluetongue surveillance in Ibaraki virus (IBV)-endemic areas because IBV-positive serum samples may result in false-positive bluetongue agar gel immunodiffusion (AGID) test reactions [65]. Because South Korea is an IBV-endemic region, the BTV VP7 Antibody Test Kit (IDEXX cELISA; IDEXX Laboratories, Inc., Institute Pourquier, Montpellier, France) was used to identify the presence of antibodies against BTV in the serum samples. BTV VP7 is the major structural protein of the inner core and contains group-specific antigenic determinants [66]. Therefore, most diagnostic methods for BTV-specific antibody detection have been based on BTV VP7 [67–75]. The assays were performed according to the manufacturer’s instructions. The specificity and sensitivity of the IDEXX cELISA kit are 100 and 82.8%, as stated by the manufacturer, respectively [76]. A herd was defined as “positive” when at least one seropositive sample was present. The seroprevalence rates and 95% confidence intervals were calculated using the program ‘Survey Toolbox for Livestock Diseases’ (Ausvet, Australia). The serotype-specific serum neutralization test (SNT) was used to confirm the positive ELISA results. Positive and negative controls for the SNT were obtained from the Institute for Animal Health, Pirbright, United Kingdom. The microtiter neutralization method was used in this study according to the Manual of Diagnostic Tests and Vaccines for Terrestrial Animals [77]. The BTV strains were used in this study as follows: RSArrr/01 (serotype 1), RSArrr/02 (serotype 2), RSArrr/03 (serotype 3), RSArrr/04 (serotype 4), RSArrr/05 (serotype 5), RSArrr/06 (serotype 6), RSArrr/07 (serotype 7), RSArrr/08 (serotype 8), RSArrr/09 (serotype 9), RSArrr/10 (serotype 10), RSArrr/11 (serotype 11), RSArrr/12 (serotype 12), RSArrr/13 (serotype 13), RSArrr/14 (serotype 14), RSArrr/15 (serotype 15), RSArrr/16 (serotype 16), RSArrr/17 (serotype 17), RSArrr/18 (serotype 18), RSArrr/19 (serotype 19), RSArrr/20 (serotype 20), RSArrr/21 (serotype 21), RSArrr/22 (serotype 22), RSArrr/23 (serotype 23), and RSArrr/24 (serotype 24). Briefly, approximately 100 TCID_50_ (50% tissue culture infective dose) of the standard or untyped virus was added to a volume of 50 μl to test the wells of a flat-bottomed microtiter plate and was mixed with an equal volume of standard antiserum that had been serially diluted in tissue culture medium. Approximately 10^4^ Vero cells (CCL-81, American Type Culture Collection, Manassas, VA, USA) were added per well in a volume of 100 μl and were assessed after incubation for 4–6 days using an inverted microscope (Olympus, Tokyo, Japan). The wells were scored for the degree of cytopathic effects observed.

### RT-PCR for BTV RNA detection

A total of 466 blood samples was tested for the presence of BTV RNA using non-serotype-specific reverse transcription PCR targeting a conserved region within the VP3 gene of the BTV genome according to the method described by Yeh et al. [78]. It is known that RNA segment 3 of BTV encodes a serogroup-reactive protein, VP3, and that VP3 sequences are often used for the genetic characterization of BTV serotypes worldwide [79–81]. Briefly, total nucleic acids were extracted from 400 μl of whole blood. Automated extraction was performed using a BioRobot M48 workstation apparatus (Qiagen, GmbH, Hilden, Germany) with a MagAttract Virus Mini M48 kit (Qiagen). Nucleic acids were recovered in 50 μl of elution buffer. RT-PCR was performed using a one-step RT-PCR kit (Qiagen). The reactions were prepared in a volume of 25 μl containing 2 μl of RNA, 1× buffer [Tris-Cl, KCl, (NH_4_)_2_SO_4_], 2.5 mM MgCl_2_, 0.2 mM deoxynucleoside triphosphates, 0.4 μM aliquots of each of the specific primers, 5 U of RNase inhibitor (Intron Biotechnology, Korea), and 1 μl of enzyme mix (Omniscript and Sensiscript RTs, HotStartTaq DNA polymerase; Qiagen). Reverse transcription amplification was accomplished in one step using the following optimized incubation program: 30 min at 50 °C; 15 min at 95 °C; 40 cycles of 94 °C for 30 s, 61 °C for 90 s, and 72 °C for 50 s; and 1 min at 72 °C. RT-PCR amplifications were performed using an Eppendorf MasterCycler gradient thermal cycler (Eppendorf, Germany). The RT-PCR amplification products (5 μl) were analyzed by gel electrophoresis on a 3% agarose gel containing 0.5 μg of ethidium bromide/ml. For the identification and differentiation of bluetongue virus serotypes, previously published serotype-specific RT-PCR [82] was performed using BTV RNA-positive samples identified by RT-PCR to detect pan-BTV. The positive control for the BTV serotype RNAs was prepared according to a method previously described by Yeh et al. [78].

### Statistical analysis and risk factor analysis

The true prevalence (TP) was calculated from the apparent prevalence (AP) using the Rogan and Gladen equation. The formula for the calculation is as follows: [83]

$$ \mathrm{TP}=\left(\mathrm{AP}+\mathrm{Sp}-1\right)/\left(\mathrm{Se}+\mathrm{Sp}-1\right). $$

The TP and Blaker’s 95% confidence intervals (CI) [84] were calculated using the Epitools epidemiological calculator [85]. Clustering of disease was explored using the analysis of variance (ANOVA) estimator of the intra-class correlation. A measure for agreement in serologic status between animals within a herd is given by the intra-class correlation coefficient μ. The intra-class correlation coefficient (minimum 0, maximum 1) was estimated using analysis of variance, with herd as the independent variable and the serologic status of individual animals (seropositive or seronegative) as the dependent variable [86]. In this study, univariable and multivariable analyses were performed. The following individual exposure variables were considered for the univariable and multivariable analyses: land use (agricultural, woodland and semi-natural and urban areas, according to KAHIS), the population sizes of the herds, and the cattle population size inside a 1 km-buffer around the sampling farm (hereafter called cattle density). The radius size was chosen considering the most likely Culicoides flying range < 1 km [87]. The herd size and number of animals were divided into classes following the tertile classification method: herd size (≤5, 6–30, ≥31), cattle density (≤10, 11–20, ≥21), and adult/calf ratios ≤1.0, 1.1–2.0, ≥2.1). The animals were also classified into three age groups based on tooth replacement and livestock owner questionnaires: juveniles (between 6 months and 1 year old), sub-adults (between 1 and 2 years old), and adults (> 2 years old). Regional risk factors, such as geographic location and local seropositivity risk factors based on livestock owner questionnaires—e.g., reproductive problems, including abortion—were also investigated. A logistic regression model was used to check the association of the animal seropositivity outcome with potential risk factors. The effect of the exposure variables on individual seropositivity was analyzed using univariable logistic regression models, and the variables in the univariable analysis were screened for pair-wise collinearity or associations using Pearson’s correlation coefficient or the chi-squared test for continuous or categorized variables, respectively. The strength of association was calculated using odds ratios at 95% CI. A *p* value < 0.05 was considered to be statistically significant. All statistical analyses were performed using the statistical software SPSS Statistics version 25 (IBM Corp., Armonk, NY, USA).

## Data Availability

All data generated or analyzed during the study are included in this published article. The datasets used and/or analyzed in the current study are available from the corresponding author on reasonable request. Individual farm information may be protected or not provided by privacy and security concerns.

## Abbreviations

BTV: Bluetongue virus
cELISA: Competitive enzyme-linked immunosorbent assay
IBV: Ibaraki virus
OIE: World Organization for Animal Health
